# Discovery of Non-Peptide GLP-1 Positive Allosteric Modulators from Natural Products: Virtual Screening, Molecular Dynamics, ADMET Profiling, Repurposing, and Chemical Scaffolds Identification

**DOI:** 10.3390/pharmaceutics16121607

**Published:** 2024-12-17

**Authors:** Mohamed S. Gomaa, Mansour S. Alturki, Nada Tawfeeq, Dania A. Hussein, Faheem H. Pottoo, Abdulaziz H. Al Khzem, Mohammad Sarafroz, Samar Abubshait

**Affiliations:** 1 Department of Pharmaceutical Chemistry, College of Clinical Pharmacy, Imam Abdulrahman Bin Faisal University, P.O. Box 1982, Dammam 31441, Saudi Arabia; msalturki@iau.edu.sa (M.S.A.); nztawfeeq@iau.edu.sa (N.T.); ahalkhzem@iau.edu.sa (A.H.A.K.); mskausar@iau.edu.sa (M.S.); 2 Department of Pharmacology, College of Clinical Pharmacy, Imam Abdulrahman Bin Faisal University, P.O. Box 1982, Dammam 31441, Saudi Arabia; dahussein@iau.edu.sa (D.A.H.); fhpottoo@iau.edu.sa (F.H.P.); 3 Department of Chemistry, College of Science, Imam Abdulrahman Bin Faisal University, P.O. Box 1982, Dammam 31441, Saudi Arabia; sabubshait@iau.edu.sa

**Keywords:** GLP-1, natural products, virtual screening, molecular dynamics, oral therapy, repurposing, chemical scaffolds

## Abstract

**Background/Objectives:** Glucagon-like peptide-1 (GLP-1) receptor is currently one of the most explored targets exploited for the management of diabetes and obesity, with many aspects of its mechanisms behind cardiovascular protection yet to be fully elucidated. Research dedicated towards the development of oral GLP-1 therapy and non-peptide ligands with broader clinical applications is crucial towards unveiling the full therapeutic capacity of this potent class of medicines. **Methods:** This study describes the virtual screening of a natural product database consisting of 695,133 compounds for positive GLP-1 allosteric modulation. The database, obtained from the Coconut website, was filtered according to a set of physicochemical descriptors, then was shape screened against the crystal ligand conformation. This filtered database consisting of 26,325 compounds was used for virtual screening against the GLP-1 allosteric site. **Results:** The results identified ten best hits with the XP score ranging from −9.6 to −7.6 and MM-GBSA scores ranging from −50.8 to −32.4 and another 58 hits from docked pose filter and a second round of XP docking and MM-GBSA calculation followed by molecular dynamics. The analysis of results identified hits from various natural products (NPs) classes, to whom attributed antidiabetic and anti-obesity effects have been previously reported. The results also pointed to β-lactam antibiotics that may be evaluated in drug repurposing studies for off-target effects. The calculated ADMET properties for those hits revealed suitable profiles for further development in terms of bioavailability and toxicity. **Conclusions:** The current study identified several NPs as potential GLP-1 positive allosteric modulators and revealed common structural scaffolds including peptidomimetics, lactams, coumarins, and sulfonamides with peptidomimetics being the most prominent especially in indole and coumarin cores.

## 1. Introduction

Diabetes mellitus (DM) is a chronic metabolic disorder that is characterized by hyperglycemia. It arises due to defects in insulin secretion, action, or a combination of both [1]. DM has been classified into four types. The most prevalent type is type 2 DM (T2DM), which accounts for approximately 90% of all diabetes cases [2]. T2DM is characterized by insulin resistance, where insulin is produced but the body’s cells do not respond effectively to it [1,3]. DM has been associated with increased associative morbidity and mortality, often as a consequence of macro- and microvascular complications. DM patients are often subject to a decline in overall health status and reduced quality of life due to the development of subsequent co-morbidities [4,5]. It is anticipated that DM will rank as the seventh leading cause of death worldwide by 2030 [6]. According to The International Diabetes Federation (IDF), approximately 250 million individuals globally are currently affected by diabetes, with projections indicating that this figure could rise to 435 million by the year 2030 [4]. The pathophysiological development of T2DM is intertwined with obesity and together they represent one of most significant threats to public health globally. The steady global increase in DM is largely driven by the rising obesity rates. Recent studies indicate that the prevalence of obesity has nearly tripled worldwide over the past 40 years, with a notable increase in the incidence of T2D associated with this trend [4,7]. In 2024, the prevalence of obesity among adults was reported to be alarmingly high, with estimates suggesting that over one billion individuals are affected globally [8].

Saudi Arabia has among the highest obesity and diabetes rates in the Middle East. Recent data indicate that more than a third of adults are classified as obese and over 20% of the Saudi population suffers from DM, with T2DM being most prevalent [9,10]. Estimates indicate that approximately 7 million individuals in the population are living with diabetes, while nearly 3 million are classified as pre-diabetic [11]. The World Health Organization (WHO) has highlighted that Saudi Arabia ranks as the second highest in the Middle East and seventh globally in terms of diabetes prevalence [12]. Projections suggest that these rates are expected to more than double by the year 2030 [13].

Consequently, these rising prevalence rates necessitate comprehensive and focused intervention strategies to mitigate both obesity and DM prevalence. The management of DM involves a range of therapeutic agents designed to achieve glycemic control and reduce blood sugar levels. These agents can be categorized into several classes, including insulin agents, insulin secretion-promoting agents, agents that enhance insulin sensitivity, and gastrointestinal glucose absorption inhibitors [14]. While these medications are effective in managing blood glucose levels, they may also be associated with several adverse effects, including hypoglycemia, weight gain, edema, gastrointestinal disturbances, and in some cases, a lack of efficacy [14].

Recently, significant interest has been directed towards glucagon-like peptide-1 (GLP-1), an insulinotropic gastrointestinal hormone secreted mainly by intestinal L-cells in response to nutrients [15]. GLP-1 receptor agonists (GLP-1 RAs) are presented as a promising and innovative method for managing diabetes as they are the first antidiabetic agent without the drawbacks of hypoglycemia or weight gain [16]. These drugs work by mimicking the effects of the GLP-1 hormone and help regulate blood sugar levels by stimulating insulin release, slowing gastric emptying, and reducing appetite [16,17]. Few GLP-1 RAs have been approved by the U.S. Food and Drug Administration (FDA) for the treatment of T2DM and, in some cases, obesity. Some of the most well-known GLP-1 RAs include exenatide, liraglutide, semaglutide, and dulaglutide [18,19,20,21]. Among these, semaglutide and liraglutide have both received FDA approval for weight management in addition to diabetes control [22,23,24]. The dual role of GLP-1 RAs in controlling blood sugar and promoting weight loss has made them a popular option for managing T2DM and obesity, conditions that are often co-existing and mutually aggravating.

Presently, GLP-1 RAs are mostly administered via subcutaneous injections as the oral delivery of peptide therapeutics is limited due to challenges in bioavailability [25]. The oral GLP-1 RAs are rapidly degraded by dipeptidyl peptidase IV (DPP-IV) and shown to have a short half-life in vivo [26]. The search for oral GLP-1 therapies is necessary, as they offer a more convenient alternative that has the potential to enhance patient acceptance and adherence compared to traditional injectable treatments. Multiple attempts have been made to overcome the bioavailability challenge. For example, the oral form of semaglutide was approved in 2019 by the FDA as the first oral GLP-1 RAs for the management of T2DM [27]. It is co-formulated with the absorption enhancer sodium N-(8-[2-hydroxybenzoyl] amino) caprylate (SNAC). Oral semaglutide has demonstrated similar benefits to injectable forms, including effective blood glucose control, weight reduction, and a low risk of hypoglycemia [28,29]. However, specific administration guidelines must be implemented. It must be taken in the morning, on an empty stomach, at least 30 min before any food, beverage, or other oral medications, and with a maximum of 120 mL of water to ensure adequate drug absorption [27,28,30]. Still, its oral bioavailability is less than 1% [27,31]. Moreover, it may induce slightly greater severity of nausea and gastrointestinal side effects compared to injectable GLP-1 RAs [32].

An alternative approach to overcome the absorption inconvenience is by developing non-peptide GLP-1 RAs. In recent years, some non-peptidic orthosteric GLP-1 RAs that share the same binding sites as the endogenous ligand have been described [33,34,35]. One of the first reported non-peptide GLP-1 RAs is the cyclobutane derivative, Boc5 [36]. However, Boc5 showed poor drug-like properties and has not been further developed as an oral drug [37]. Since then, other non-peptide GLP-1 RAs such as LY3502970 (orforglipron), TT-OAD2, and PF-06882961 (OWL-833) succeeded in entering clinical trials [31,38,39,40,41]. Despite extensive research and development efforts, none of the candidates have successfully made it to market thus far. The high conservation of the orthosteric binding sites for GLP-1 presents a significant challenge in achieving high selectivity for GLP-1 receptors. Additionally, the elongated structure of the GLP-1 binding groove, coupled with the presence of numerous interactions along this groove, complicates the task of mimicking the peptide–receptor interactions using non-peptide molecules [42].

Positive allosteric modulation represents another valuable strategy for targeting GLP-1 R. Positive allosteric modulators (PAMs) bind to an allosteric site rather than the orthosteric binding site leading to enhance the binding affinity and/or efficacy of natural agonists at their respective receptors [43]. PAMs offer a distinctive therapeutic opportunity due to their ability to demonstrate probe dependence and biased signaling [44]. Allosteric sites exhibit less conservation compared to orthosteric sites, and targeting these sites can enhance subtype selectivity while minimizing side effects [45]. Among the PAMs studied for the GLP-1 receptor, the compounds developed by Novo Nordisk and Eli Lilly (BETP, also known as compound **B**) and its derivatives are the most extensively researched [42]. Recently, they reported the identification of PAM of the GLP-1R that engages in a cooperative interaction with both the receptor and GLP-1 [46]. This discovery exposes a new druggable pocket for a small molecule and presents exciting opportunities for developing oral agents that can activate GLP-1R [46].

In this study, we virtually screen natural products (NPs) databases to find non-peptide GLP-1 allosteric modulators that can bind to this druggable pocket. Given the accessibility and cost-effectiveness of NP, these compounds may provide a solution to the challenge of identifying an oral GLP-1 therapeutic agent. Medicinal plants play a significant role in the management of various diseases worldwide, including DM. According to literature, around 400 plants and 700 plant-based formulations have been documented globally for the management of DM [47,48,49]. Moreover, recent studies have identified some medicinal plants with modulatory activity on GLP-1 [15,48,49,50,51]. At the present time, the use of medicinal plants is increasingly recognized and accepted, not just seen as an alternative approach, but as a mainstream option, due to the extensive research and the implementation of supportive policies [52].

## 2. Materials and Methods

### 2.1. Materials and Software

The molecular modeling software Maestro by Schrödinger (Version 2024-4 software release) [53] (RDIA Grant 12990-iau-2023-iau-R-3-1-HW: P.O. 6947 License key: 03cb87b8-723c-4fec-9b8c-8a58137d7a76) was used for computational studies. The desktop workstation was equipped with Intel^®^ Core™ i7-10700F Processor, Linux Ubuntu 22.10 operating system and an RTX 5000 graphics card.

### 2.2. Database Preparation

The natural products (NPs) database consisting of 695133 NPs was downloaded from the Coconut website “https://coconut.naturalproducts.net/ (accessed on 20 June 2024)”. The obtained structures were filtered using Schrödinger’s Canva based on their physicochemical descriptors including MW, logP, number of rings, total heavy atoms count, total charge, number of H-donors, number of H-acceptors, number of rotatable bonds, total charge, and QED score. Final database filtration comprised no violation of rule of 5 [54] and a natural product-like (NPL) score <2 [55].

### 2.3. Shape Screening

Shape screening was performed using the shape screening tool on Schrödinger Maestro. The filtered database was first energy minimized in Maestro using the OPLS3 force field and a default value of 0.30 Å for rmsd for non-hydrogen atoms and then subjected to shape screening using the structure of the crystal ligand as the reference structure. The typed pharmacophore technique was used for volume scoring. This technique evaluates the compounds based on their pharmacophore features. The shape similarity (SS) score was used to rank the compounds and a threshold of 0.3 was utilized to further filter the database for virtual screening [56].

### 2.4. Crystal Structures

The Cryo-EM structure of the GLP-1 receptor in complex with G protein, GLP-1 peptide, and a positive allosteric modulator (PDB ID: 6VCB) were extracted from the Research Collaboratory for Structural Bioinformatics (RCSB) Protein Data Bank (PDB) [57,58].

### 2.5. Protein Preparation

The protein structure was prepared for docking using the Protein Preparation Workflow on Maestro. The preparation and minimization process were carried out at a pH of 7.4, and with adjusting ionization states. Polar hydrogens were added, and non-essential water molecules were deleted from the structures. Finally, the receptors were optimized using OPLS3 force field. In the final stage, the optimization and minimization on the ligand–protein complexes were carried out with the OPLS3 force field and the default value for rmsd of 0.30 Å for non-hydrogen atoms was used [59]. The receptor grids were then created at the center of the bound ligand utilizing a 1.00 van der Waals (vdW) radius. The scaling factor for vdW radius was used, along with a cutoff of 0.25 for partial charges. The binding sites were contained inside a grid box of 20 Å^3^ using default parameters and without any constraints.

### 2.6. Ligand Library Preparation

The final filtered ligands were prepared using the built-in LigPrep tool in the virtual screening workflow available in Maestro. The ligands’ three-dimensional structures were created by adding missing hydrogens and generating the most likely ionization states at a pH of 7 ± 2 using Epik. Ligands’ geometry was then optimized with the OPLS3 force field with tautomer generation, desalting and producing 32 isomers per ligand at most [59]. The produced conformations represented the initial input structures for the virtual screening workflow.

### 2.7. Validation of Molecular Docking

The validation of the molecular docking method was performed to evaluate the accuracy of Maestro Glide to predict docking poses for the studied protein [60,61]. The crystal ligand was docked into GLP-1 receptor using the same criteria used for library screening. The docked pose with the lowest binding energy was aligned with the crystal structure conformation using Maestro’s structure superimposition feature. The root mean square deviation (RMSD) of the alignment was then calculated.

### 2.8. Virtual Screening

The virtual screening workflow involved three steps: high throughput virtual screening (HTVS), standard precision docking (SP), and extra precision docking (XP). For each docking protocol, flexible docking was selected with performing post-docking minimization, generating three poses per compound and holding 10% of the best scoring compounds. No more filters or constraints were applied in the docking process. The specific parameters used were a van der Waals (vdw) radius scaling factor of 0.80 and a partial charge cut-off of 0.15. The XP score was utilized to rank ligands and determine hit molecules. Molecular mechanics–generalized Born surface area (MM-GBSA) binding free energy calculation was applied from the virtual screening workflow to estimate the binding affinity for the top hit compounds using the Prime MM-GBSA module.

### 2.9. Docked Poses Filter

The docking output from the SP and XP docking was subjected to docked poses filtration based on previous experimental findings in the crystal structure using the pose filter tool in Maestro. The filtration criteria utilized were selected to must be all fulfilled and included contact with Leu 142 within 5 Å, π-π contact with Tyr 145 within 5 Å, H bond with Lys 202 within 2.5 Å, maximum H bond distance of 2.5 Å, minimum H bond-donor angle of 90 and H bond-acceptor angle of 60, and contact with Phe 12, Val 16, and Leu 20.

### 2.10. Molecular Dynamics

Molecular dynamic (MD) simulation studies were conducted using the Desmond Module on Schrödinger’s Maestro platform as previously described. In brief, the ligand–protein complex in its optimal docked configuration was first minimized using the Protein Preparation Wizard and prepared for MD simulation using the system builder application of Desmond. The simulation environment generated contained a water-based solvent system: the TIP3P water model. An orthorhombic simulation box with a 10 Å buffer parameter from the protein surface was generated; the entire system was neutralized by calculating and adding the required number of counter ions and 0.15 M NaCl in order to attain isosmotic conditions. MD simulation was carried out at a temperature and atmospheric pressure of 300 K and 1.013 bar, respectively. The simulation was run for a total of 100 nanoseconds (1001 frames were saved in order to compile the trajectory). Analysis was run and results presented using the simulation interaction diagram tool of Desmond.

### 2.11. ADMET Profiling

We used the pkCSM web server (http://biosig.unimelb.edu.au/pkcsm/prediction accessed on 11 November 2024) [62], in predicting the ADMET absorption, distribution, metabolism, excretion, and toxicity descriptors together with drug-likeness properties for the finally selected potential inhibitors. A total of eight molecular descriptors were generated in calculating the ADMET attributes in the potential KHK hits. In addition, Swiss ADME www.swissadme.ch/ accessed 11 November 2024 [63] was implemented for the calculation of the physicochemical properties, medicinal chemistry aspects, and drug-likeness attributes.

## 3. Results and Discussion

A combined structure-based and ligand-based approach was used to identify ligands that bind to the GLP-1 receptor allosteric site and enhance its activity with the aim of discovering novel natural molecules that would assist in the management of diabetes and obesity via GLP-1 modulation activity. The drug discovery protocol focuses on the identification of orally active drug-like molecules to address the current limitation of injection therapy. The results were analyzed to identify potential candidates for repurposing and to elucidate common structure scaffolds for modulation. The results were also surveyed for natural products with previously reported antidiabetic and anti-obesity effects to which a GLP-1 receptor modulation would be postulated.

### 3.1. Database Preparation for Virtual Screening

#### 3.1.1. Physicochemical Parameters and Drug-Likeness

**Figure 1:**
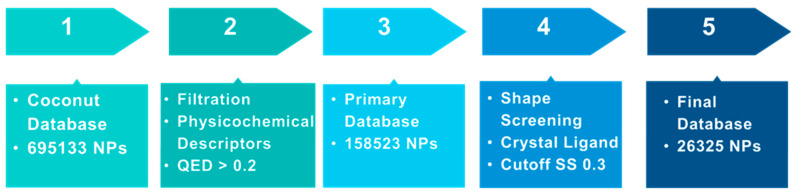
Filtration protocol for the Coconut natural products database.

The main goal of the current research is to identify orally active GLP-1 positive allosteric modulators from natural sources. A filtration protocol was designed with a focus on drug-like property calculations (Figure 1). The quantitative estimation of the drug-likeness (QED) model developed in FAFDrugs4 combines several rules and principles describing a target drug’s physicochemical properties, including rule of 3, rule of 5, Zinc, CNS, and respiratory drug-likeness principles, combined together with a statistical analysis of approved drugs [64]. The resulting chemical space was found to accommodate up to 90% of these oral drugs and thereby validated the filter thresholds. The Coconut database is a comprehensive dataset of elucidated and predicted natural products (NPs) obtained from public databases, combined with an intuitive web interface [65,66]. It incorporates 63 databases of NPs that include among others, ChemSpider NPs, NCI development therapeutics, PubChem NPS, Zinc NPs, and the NPAtlas. This huge database contains 695,133 NPs on its latest release in 2024. The complete database was first downloaded from the Coconut website and was then filtered using Schrödinger’s Canva through escaping duplicate structures and applying a set of physicochemical descriptors that were initially incorporated in the database, including MW 100–600, logP −3–6, number of rings < 6, total heavy atoms count < 50, total charge −4–4, number of H-donors < 7, number of H-acceptors < 12, number of rotatable bonds < 11, and total charge −4–4. QED integrates eight physicochemical properties: molecular weight, LogP, H-bond donors, H-bond acceptors, charge, aromaticity, stereochemistry, and solubility in a score ranging from 0–1. The molecule is considered more drug-like when its QED score is closer to 1 [67]. A QED score > 0.2 with no violation of the rule of 5 permitted and a natural product-like (NPL) score < 2 were applied for final database filtration. A confined database of 158,523 compounds was obtained and was then subjected to further refinement using shape screening.

#### 3.1.2. Shape Screening

**Figure 2:**
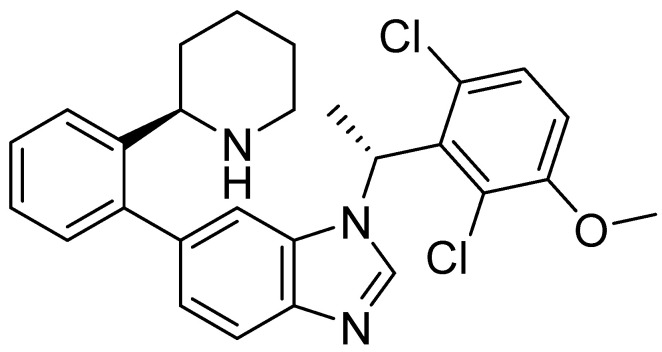
Chemical structure of GLP-1 co-crystallized ligands positive allosteric modulator used in the shape screening.

The resulting database containing 158,523 natural products was subjected to a shape screening analysis on Schrödinger’s Canva against the experimental GLP-1 co-crystallized allosteric modulator (Figure 2). Ligands were first minimized and then used as a query in the 3D alignment with the filtered database entries with conformers generation. The results of the shape screening were filtered with a cutoff shape similarity index below 0.3. This resulted in a final database containing 26,325 compounds to be used for virtual screening against the allosteric site of the GLP-1 receptor.

### 3.2. Virtual Screening

**Figure 3:**
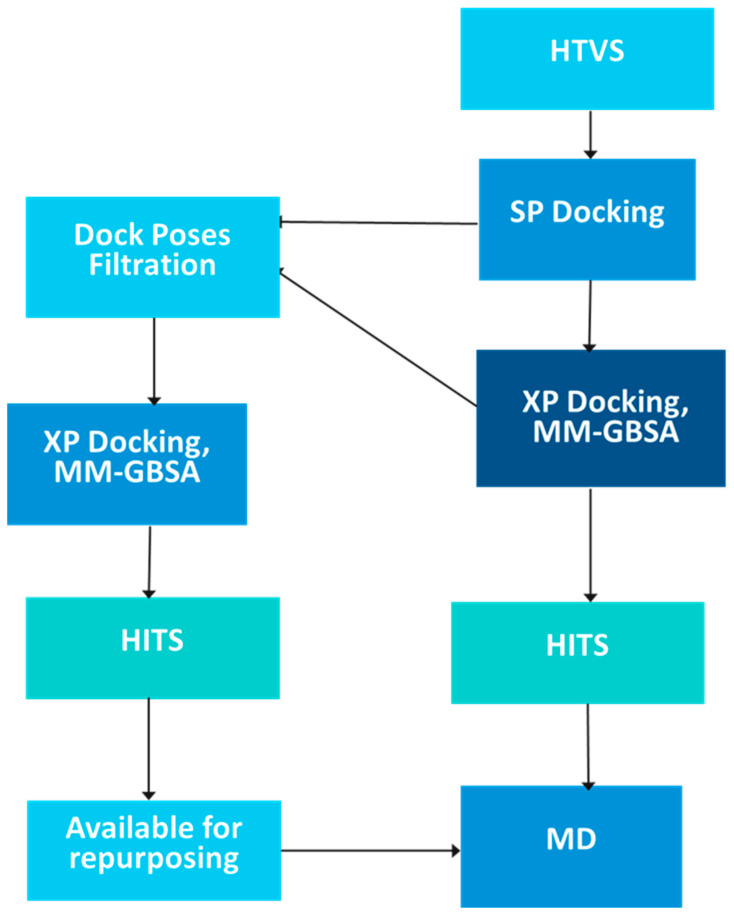
Hit identification protocol.

The virtual screening of the final database involved three stages including high throughput screening (HTVS), standard precision (SP), and extra precision (XP) docking. The protocol used a flexible docking method which incorporated post-docking energy minimization and was set to keep 10% of the best scoring compounds in each phase while retaining scoring hierarchy. The final output after the XP docking stage included 136 natural products. These compounds were further subjected to a second round of XP docking using the same criteria to produce a final hits list of 10 natural products. The binding energies (MM-GBSA) were calculated for the best 10 hits and were correlated with their XP score (Figure 3). The docking protocol was initially validated by calculating the RMSD of the crystal and docked poses of the co-crystallized ligand that was found to be 1.77 Å, a figure that establishes a good accuracy for glide to predict binding to the GLP-1 allosteric site.

The XP scores ranged from −9.6 to −7.6, with a maximum and minimum score difference of two units. Compared to the XP score (−3.3) of the crystal ligands, the identified hits showed better affinity to the GLP-1 allosteric site based on their docking scores. The calculated binding energies (MM-BBSA DG Bind) ranged from −50.8 to −32.4 and showed acceptable correlation with the XP scores especially for hits **1**, **5**, **6**, **7**, **8** that have SP scores below −8.0 and MM-BBSA DG Bind below −40.0 (Table 1). Given the fact that the hits are not a congeneric series, the ranking based on MM-GBSA DG Bind are not expected to perfectly correspond with the ranking based on docking scores and experimental binding affinity. MM-GBSA is more reliable in prioritizing compounds for experimental testing compared to alternative docking calculations [68]. It represents the calculated total free energies of the protein–ligand complex which is the sum of the van der Waals, electrostatic, General Born solvation, and surface area energies [69].

The crystal ligand binds in a pocket formed by residues within the interface between TM1 and TM2 and forms van der Waals (vdW) contacts with Leu 142 and π-π stacking with Tyr 145 [46]. In a mutagenesis study by Bueno et al. [46], Leu 142 and Tyr 145 single mutants reduced the potentiation effect of the allosteric modulator on GLP-1 binding to its receptor in cAMP assays. The Lys 202 mutant also showed a slight reduction in the potentiation effect. Triple mutation (Leu 142, Tyr 145, Lys 202) completely abolished the positive modulation activity and indicated π–π stacking is the primary interaction between the ligand and Tyr 145 [46].

In addition to the ligand–receptor interactions, ligand–GLP-1 contacts were also found to be detrimental. Establishing contacts with Phe 12, Val 16, and Leu 20 of the GLP-1 peptide is of great importance. Such interaction with Phe 12 of the GLP-1 peptide stabilizes its vdW contact with Leu 384 and Leu 388 of the receptor. The vdW interactions between the peptide and ligand are crucial for affinity as the affinity of the mutant peptide GLP-1 Phe 12, or Val 16, or Leu 20 can only be weakly potentiated by the allosteric modulator when compared to native GLP-1 [46].

The analysis of the binding pattern and interactions with the key residues revealed that the hits were able to bind the key residues Leu 142, Tyr 145, and Lys 202, combined or selected ones. The analysis also uncovered the importance of other important residues in the allosteric pocket that would increase the binding affinity of potential ligands and revealed additional important binding interactions with residues including Ser 206, Glu 138, and Asp 198 (Table 2). Interestingly, the hit compounds were able to establish H bonds with the key residues, an interaction that was absent from the resolved crystal ligand to 3.3 Å.

**Figure 4:**
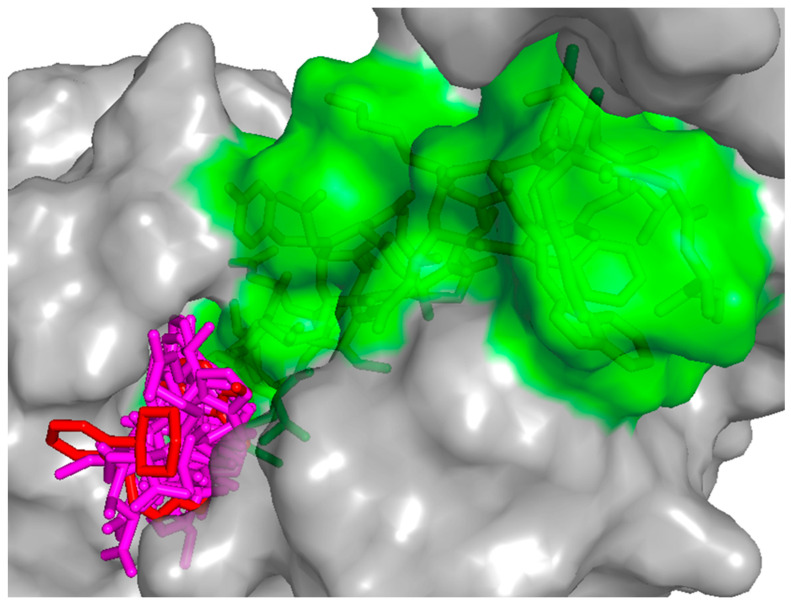
Surface representation of the overlay of the top 10 hits (magenta sticks) and the crystal ligand (red stick) in GLP-1 allosteric site (PDB ID: 6VCB). GLP-1 receptor is represented in gray surface and GLP-1 peptide in green surface.

Comparative docking of the top ten hits with the crystal ligand showed that they became deeper in the binding site and closer to the GLP-1 peptide. This resulted in better interactions with key residues in both the GLP-1 receptor and GLP-1 peptide and an overall better XP score (Figure 4).

**Figure 5:**
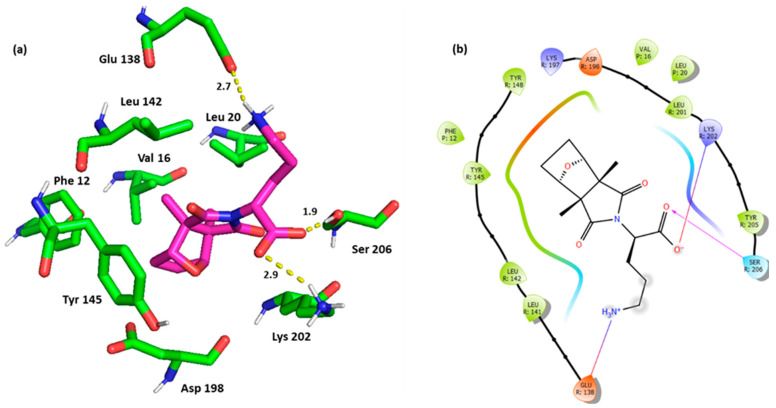
(**a**) Three-dimensional representation of the binding interactions between hit **1** and GLP-1 receptor allosteric site and GLP-1 peptide (PDB ID: 6VCB). Ligand atoms are shown as sticks (carbon atoms colored in magenta) and the key residues are shown as sticks (carbon atoms colored in green). Potential electrostatic interactions are represented as yellow dotted lines and are measured in Angstrom. (**b**) Two-dimensional ligand–protein binding interactions between hit **1** and GLP-1 receptor allosteric site and GLP-1 peptide (PDB ID: 6VCB). H bond is represented as a purple arrow and salt bridge as a blue line.

Hit **1** docking pose showed interesting, centered orientation of its fused ring that enabled vdW interactions with the receptor key residues; Leu 142, and Tyr 145 and the peptide key residues; Phe 12, Val 16, and to a lesser extent Leu 20. Other important noted electrostatic interactions involved a salt bridge with the receptor key residue Lys 202 and a H bond with Ser 206 (Figure 5).

The analysis was further extended to identify any compounds from the initial XP hits (136 compounds), SP hits (1532 compounds), that showed considerable interactions with the key residues and possess a good XP/Glide score. This was done through the docked poses filter tool on maestro. The filtration comprised a set of criteria that must be all fulfilled including contact with Leu 142 within 5 Å, π-π contact with Tyr 145 within 5 Å, H bond with Lys 202 within 2.5 Å, maximum H bond distance of 2.5 Å, minimum H bond–donor angle of 90 and H bond–acceptor angle of 60, and contact with Phe 12, Val 16, and Leu 20. The XP/SP score cutoff was set to −5.0 to ensure that other interactions are not negatively impacting the overall binding.

The shortlisted compounds were docked again in the GLP-1 allosteric site using extra precision and their binding energies were calculated to further assess their binding affinity. All compounds from the XP docking that had a score of −3.3 (crystal ligand XP score) and below with a negative binding energy are considered as final hits. This resulted in identifying 58 more hits that exhibited the spotted interactions together with fulfilling the XP score threshold and were found to be in the range −7.4 to −3.7 (Table 3). The MM-GBSA ranged from −52.77 for hit 14 to −13.02 for hit 48 which indicates favorable interactions between those hits and the GLP-1 allosteric site in terms of binding energies (see Supplementary Materials).

**Figure 6:**
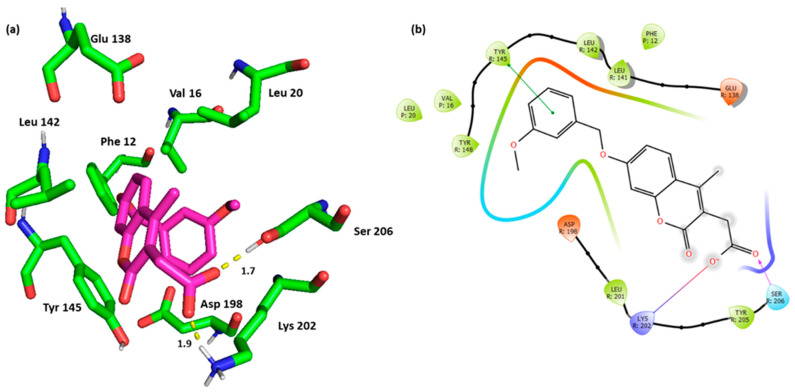
(**a**) Three-dimensional representation of the binding interactions between hit **12** and GLP-1 receptor allosteric site and GLP-1 peptide (PDB ID: 6VCB). Ligand atoms are shown as sticks (carbon atoms colored in magenta) and the key residues are shown as sticks (carbon atoms colored in green). Potential electrostatic interactions are represented as yellow dotted lines and are measured in Angstrom. (**b**) Two-dimensional ligand–protein binding interactions between hit **12** and GLP-1 receptor allosteric site and GLP-1 peptide (PDB ID: 6VCB). H bond is represented as a purple arrow, salt bridge as a blue line, and π-π stacking as a green line.

Hit **12** exhibited similar interactions to hit **1** including salt bridge with Lys 202 and H bond with Ser 206. However, unlike hit **1**, hit **12** established the sought π-π stacking with Tyr 145. The hit also maintained the vdW interactions with Leu 142, Phe 12, Val 16, and Leu 20 (Figure 6).

**Figure 7:**
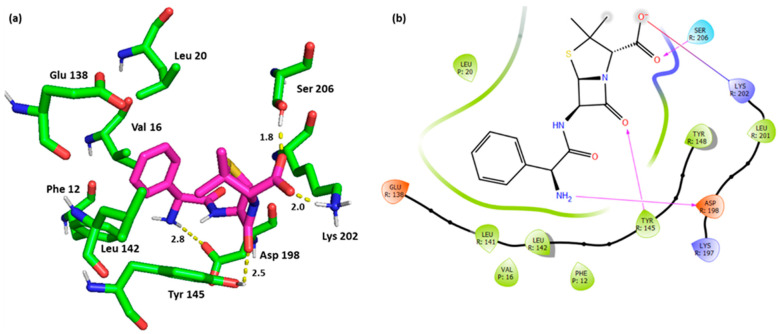
(**a**) Three-dimensional representation of the binding interactions between hit **44** (Ampicillin) and GLP-1 receptor allosteric site and GLP-1 peptide (PDB ID: 6VCB). Ligand atoms are shown as sticks (carbon atoms colored in magenta) and the key residues are shown as sticks (carbon atoms colored in green). Potential electrostatic interactions are represented as yellow dotted lines and are measured in Angstrom. (**b**) Two-dimensional ligand–protein binding interactions between hit **44** (Ampicillin) and GLP-1 receptor allosteric site and GLP-1 peptide (PDB ID: 6VCB). H bond is represented as a purple arrow, and salt bridge as a blue line.

Interestingly, Ampicillin (hit **44**) was identified as a hit molecule, being able to establish the key interactions. Ampicillin bound the key residue Tyr 145 with its β-lactam oxygen through the H bond. Although this type of interaction with Tyr 145 was not mentioned in the binding requirements of the crystal structure, more investigations need to be performed to establish its feasibility and impact. Again, the salt bridge with Ser 202 and the H bond with Ser 206 were maintained for Ampicillin. Additional H bond with Asp 198, as well as the previously discussed interactions with Leu 142, Phe 12, and Val 16 were also noted (Figure 7).

In conclusion, the current docking studies not only identified new hits for GLP-1 positive allosteric modulation, but also shed the light about the importance of Ser 206 as a crucial residue with Lys 202 and to a lesser extent Glu 138, and Asp 198 that would enhance the affinity and selectivity of potential ligands.

### 3.3. Molecular Dynamics

**Figure 8:**
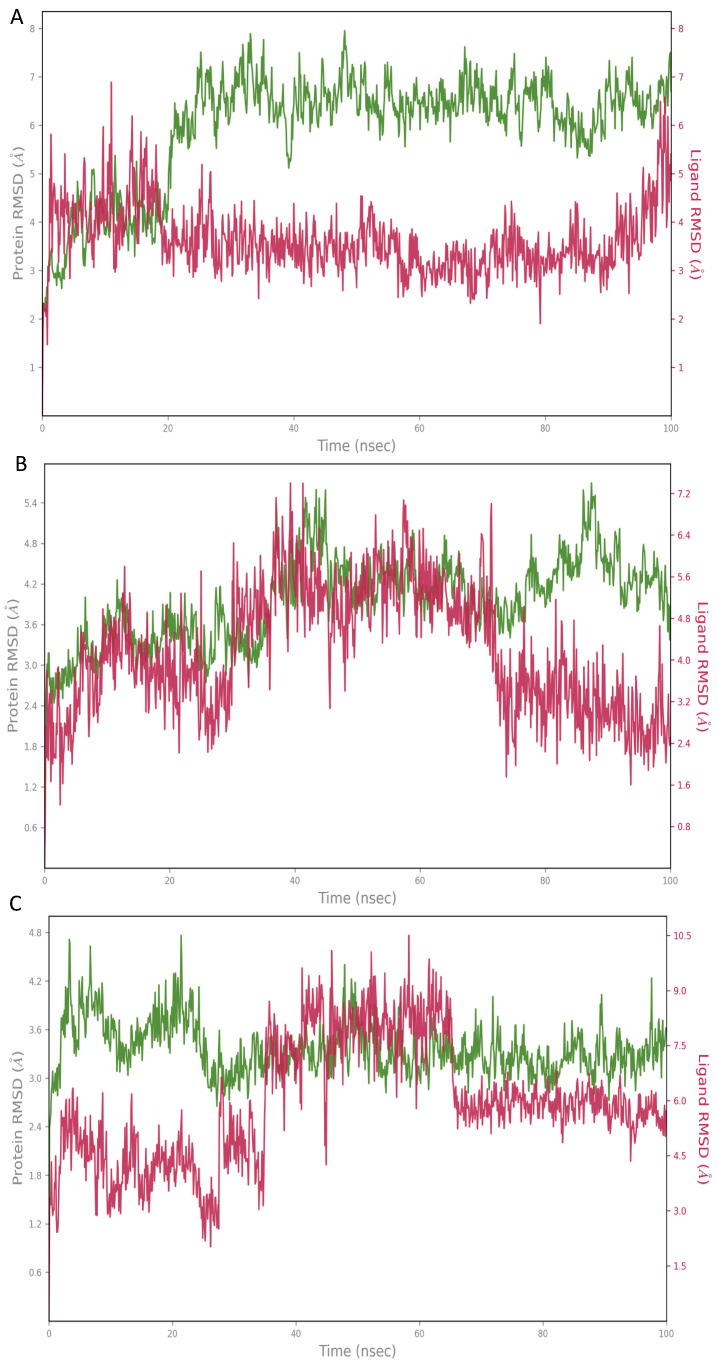
Root mean square deviation (RMSD) graphs for the hit compounds (**A**): compound **3** (CNP0086660.2), (**B**): compound **5** (CNP0039190.2), (**C**): compound **2** (CNP0549010.1). The green graph shows fluctuations in the protein backbone from the initial reference point while the red shows the ligand fluctuations. The RMSD profile of the ligand with respect to its initial fit to the protein binding pocket indicates that all ligands did not fluctuate beyond a 2–7 Å range.

**Figure 9:**
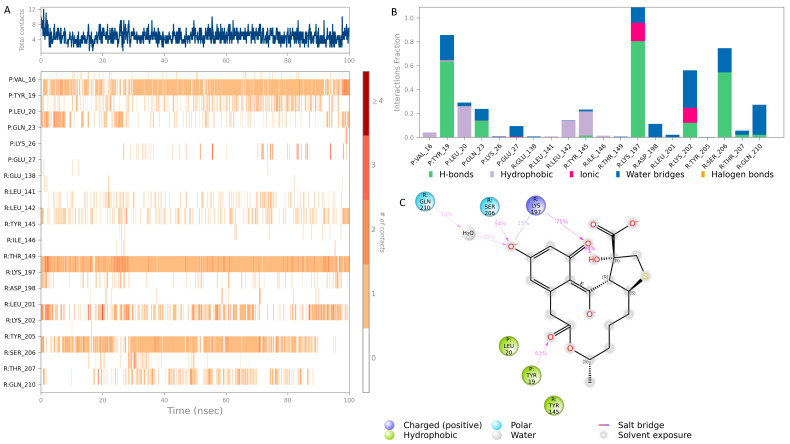
Interaction diagram of hit compound **3** (CNP0086660.2) with the GLP-1 allosteric binding pocket. (**A**) Interaction of compound **3** with residues in each trajectory frame. The depth of color indicating the higher the interaction with contact residues; (**B**) the protein–ligand contacts showing the bonding interactions fraction and the nature of the interactions; (**C**) graphical 2D illustration of compound **3** interacting with the protein residues during MD simulation.

**Figure 10:**
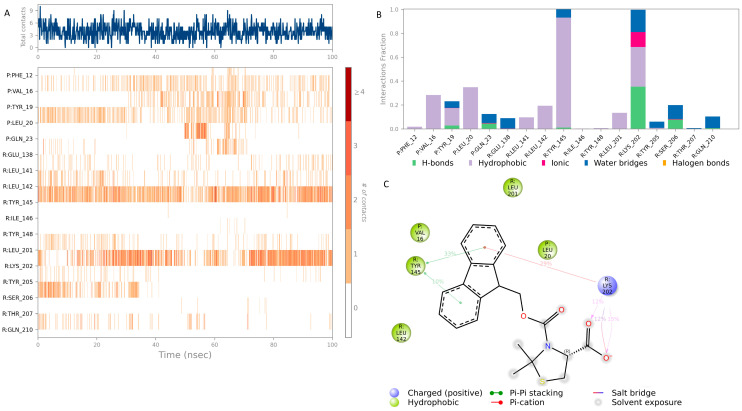
Interaction diagram of hit compound **5** (CNP0039190.2) with the GLP-1 allosteric binding pocket. (**A**) Interaction of compound **5** with residues in each trajectory frame. The depth of color indicating the higher the interaction with contact residues; (**B**) the protein–ligand contacts showing the bonding interactions fraction and the nature of the interactions; (**C**) graphical 2D illustration of compound **5** interacting with the protein residues during MD simulation.

**Figure 11:**
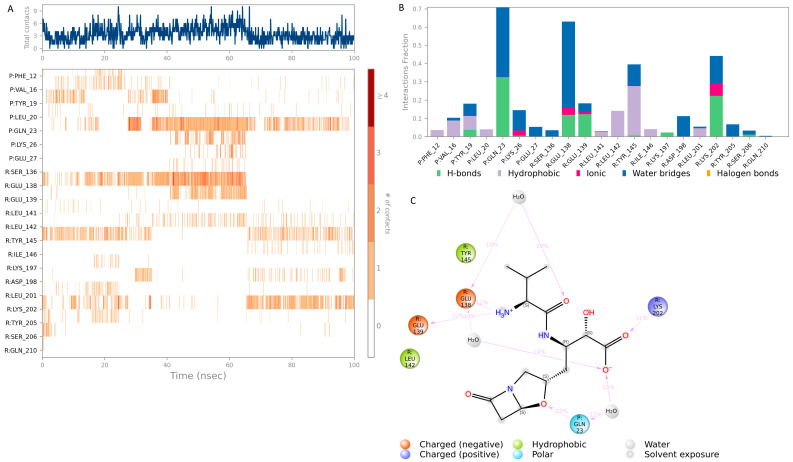
Interaction diagram of hit compound **2** (CNP0549010.1) with the GLP-1 allosteric binding pocket. (**A**) Interaction of compound **2** with residues in each trajectory frame. The depth of color indicating the higher the interaction with contact residues; (**B**) the protein–ligand contacts showing the bonding interactions fraction and the nature of the interactions; (**C**) graphical 2D illustration of compound **2** interacting with the protein residues during MD simulation.

The top hit molecules were selected based on a detailed visual analysis of the ligand–protein interaction profile. Hit compounds which had a favorable binding score and binding interactions with key residues in the binding pocket were selected for molecular dynamic simulation studies which were essential to validate the stability and strength of the binding interaction over a period of 100 nanoseconds. Of the 10 ligands used in MD simulation studies, 3 exhibited a robust and stable binding pattern within the allosteric binding pocket of the GLP-1 protein. Root mean square deviation (RMSD) plot analysis was used to measure the average displacement of atoms for a particular frame with respect to a reference frame. The analysis revealed a stable binding profile, as indicated by an average RMSD fluctuation range within 4 Å of the ligand with reference to the protein backbone throughout the simulation period. Figure 8 shows the RMSD plots for the top selected three compounds expressing the favorable binding profile with key residues in the binding pocket and also suited for drug repurposing: compound **3** (CNP0086660.2), compound **5** (CNP0039190.2), and compound **2** (CNP0549010.1). While a certain degree of fluctuation is anticipated within the simulation period of an allosteric binding pocket comprised the GLP-1 protein and its interacting peptide, the hit compounds appeared to maintain key interactions highlighted in Table 3 throughout a significant period of the 100 ns simulation period as depicted in Figure 9, Figure 10 and Figure 11. Of significance, the protein backbone in Figure 8 appears to express an expected degree of fluctuation but this too becomes stabilized within a tight range overtime. For further verification, the energy calculations were repeated for the hit compounds after molecular dynamic simulation runs using the final frame of the simulation trajectories. For the hit compounds, compound **3** and compound **2** and compound **5**, the MM-GBSA calculations were found to be −39.22, −38.18, and −48.15, respectively; these results were found to be comparable to initial energy calculations using the optimal docking orientations.

Hit 3 of the macrolide class exhibits one of the most favorable binding profiles, interacting with a number of residues of the GLP-1 allosteric binding pocket as shown in Figure 9. Notably, GLP-1 receptor binding residues Lys 197 and Ser 206 form a stable hydrogen bond with the ligand for over 70% and 50% of the simulation period, respectively, in addition to a stable hydrogen bond formed with the GLP-1 peptide residue Tyr 19 which was stable for over 60% of the simulation period. All significant interactions are represented in Figure 9A,B which highlight the residues with the strongest ligand interactions stable throughout the entire simulation period. Figure 9C depicts all significant interactions displayed by the ligand and interacting residues occurring for over 10% of the simulation period. Macrolides are a class of broad-spectrum antibiotics which are granted FDA approval for the management of a wide range of infectious disease conditions. The approval status and safety profile of this class of medications renders it particularly amenable to subsequent repurposing studies.

MD simulation results for hit **5** (CNP0039190.2) in Figure 10 show significant interactions with the key binding residues of the allosteric pocket. Key binding residues Tyr 145 forms hydrophobic interactions in the form of π-π stacking with the compound whereas a complex interaction is observed with Lys 202 and the terminal COO– group of hit **5**. The interactions with the GLP-1 receptor are observed for over 30% of the simulation period.

Figure 11 shows the binding profile of hit **2** (CNP0549010.1) with residues in the allosteric GLP-1 receptor binding pocket. Hit **2** forms moderate interactions with the key binding residues Tyr 145 and Lys 202 of the receptor binding pocket: hydrophobic and water bridge interaction with Tyr 145 and a complex hydrogen and water bridge interaction with Lys 202. Additionally, a complex hydrogen bond and water bridge interaction is observed between hit **2** and the GLP peptide residue Gln 23 for over 20% of the simulation period. While the strength of the interaction is relatively weaker compared to other hit compounds, this hit remains significant owing to its classification as a beta lactam compound. Beta lactam skeletons form the essential component of the most potent class of antibiotics clinically available. They are widely used and have an excellent safety profile making this compound a desirable target for further drug repositioning studies.

### 3.4. Literature Analysis

The hit compounds were grouped according to their natural product classification and were surveyed in the literature for their biological function to identify potential correlations with postulated GLP-1 modulation activity. The following classes have been identified and shortlisted from the literature given their favorable pharmacodynamic profile:

1. Sesquiterpenoids (Hit Numbers: **6**, **9**, **17**, **63**), particularly drimane-type sesquiterpenoids from Zygogynum pancheri (PMID: 32603660), have demonstrated significant antidiabetic and lipid-lowering effects, including α-amylase and lipase inhibition, while sesquiterpenoids from Hieracium and Pilosella species (PMID: 34358652) and Cichorium species (PMID: 38900250) exhibit broad pharmacological activities such as anti-inflammatory, antioxidant, anti-obesity, and hepatoprotective properties, emphasizing their potential as therapeutic agents in managing metabolic and chronic diseases.
2. Steroidal hormones (Hit Numbers: **7**, **24**) like dehydroepiandrosterone (DHEA) (PMID: 31586606), phytochemicals from Broussonetia species (PMID: 36014582), Brassica oleracea var. capitata (white cabbage) (PMID: 33430729), Morus alba (PMID: 36877269), Cichorium species (PMID: 38900250), and endocrine therapies (PMID: 20210723) demonstrate significant antidiabetic, anti-obesity, antioxidant, and anti-inflammatory properties, with applications ranging from traditional medicine to modern pharmacological interventions, while highlighting safety considerations such as QTc prolongation in metabolic disease management.
3. Coumarins (Hit Numbers: **11**, **12**, **35**, **66**) found in Sophora species (PMID: 34907492), Ponciri Fructus (PMID: 36615447), Hieracium and Pilosella species (PMID: 34358652), and Cichorium species (PMID: 38900250) exhibit significant pharmacological activities, including antidiabetic, anti-inflammatory, anti-obesity, antioxidant, hepatoprotective, and anticancer effects, highlighting their potential as bioactive agents in traditional medicine and modern therapeutic applications.
4. Phenylpropanoids (Hit Numbers: **16**, **35**, **41**, **50**, **60**) from the Broussonetia genus (PMID: 36014582), particularly isolated from Broussonetia papyrifera, Broussonetia kazinoki, and Broussonetia luzonica, exhibit diverse pharmacological activities, including antitumor, antioxidant, anti-inflammatory, antidiabetic, and anti-obesity effects, highlighting their significant therapeutic potential and the need for further research into their mechanisms of action and clinical applications.
5. Xanthones (Hit Number: **36**) particularly from Garcinia mangostana and Garcinia cambogia (PMIDs: 28656594, 25732350), exhibit promising pharmacological activities, including anti-obesity, antidiabetic, anti-inflammatory, and antioxidant effects, while their isoprenylated derivatives target multiple signaling pathways involved in metabolic and degenerative diseases (α-mangostin, PMID: 35904170; Anthocleista species, PMID: 26432351), positioning them as valuable bioactive compounds for developing therapies against chronic conditions.
6. Phenolic compounds (Hit Number: **50**) from diverse natural sources, including Piper species (PMID: 39277979), Vaccinium myrtillus leaves (PMID: 30052516), Hippophae rhamnoides fruit and seeds (PMID: 38358042), Prunus armeniaca leaves (PMID: 34942972), persimmon leaves (PMID: 36840285), elderberries (Sambucus nigra) (PMID: 38998923), Platycodon grandiflorum (PMID: 39072195), fermented soy products (PMID: 36014024), peanut seeds (PMID: 38000103), potatoes (Solanum tuberosum) (PMID: 35453288), fenugreek seeds (PMID: 31286789), and Origanum species (PMID: 32789910), exhibit significant antidiabetic, anti-obesity, anti-inflammatory, antioxidant, hepatoprotective, and cardioprotective effects, supporting their potential as bioactive agents in metabolic and chronic disease management through mechanisms such as enzyme inhibition, oxidative damage prevention, and modulation of inflammatory pathways.
7. Lignans (Hit Number: **60**), particularly secoisolariciresinol diglucoside (SDG) from Linum usitatissimum (flaxseed) (PMID: 33535948), exhibit diverse pharmacological activities, including antioxidant, antidiabetic, anti-obesity, anti-inflammatory, anticancer, antimicrobial, hepatoprotective, and renoprotective effects, positioning them as potent therapeutic agents for managing chronic diseases, while further research is needed to fully understand their mechanisms of action and therapeutic potential.
8. The results also identified compounds for potential repurposing with β-lactam antibiotics being the most prominent. Valclavam (hit **2**), Cyclothiocurvularin B (hit **3**), Azidocillin (hit **25**), Ampicillin (hit **44**), Metampicillin (hit **46**), Timocillin (hit **67**) were found to be, and according to this study, GLP-1 positive allosteric ligands. This finding would represent a base for future research on this class of antibiotics to prove their preclinical and clinical effectiveness in this context as well as identifying a molecular basis for their GIT-related side effects and loss of appetite.

### 3.5. Scaffolds Identification for GLP-1 Allosteric Modulation

**Figure 12:**
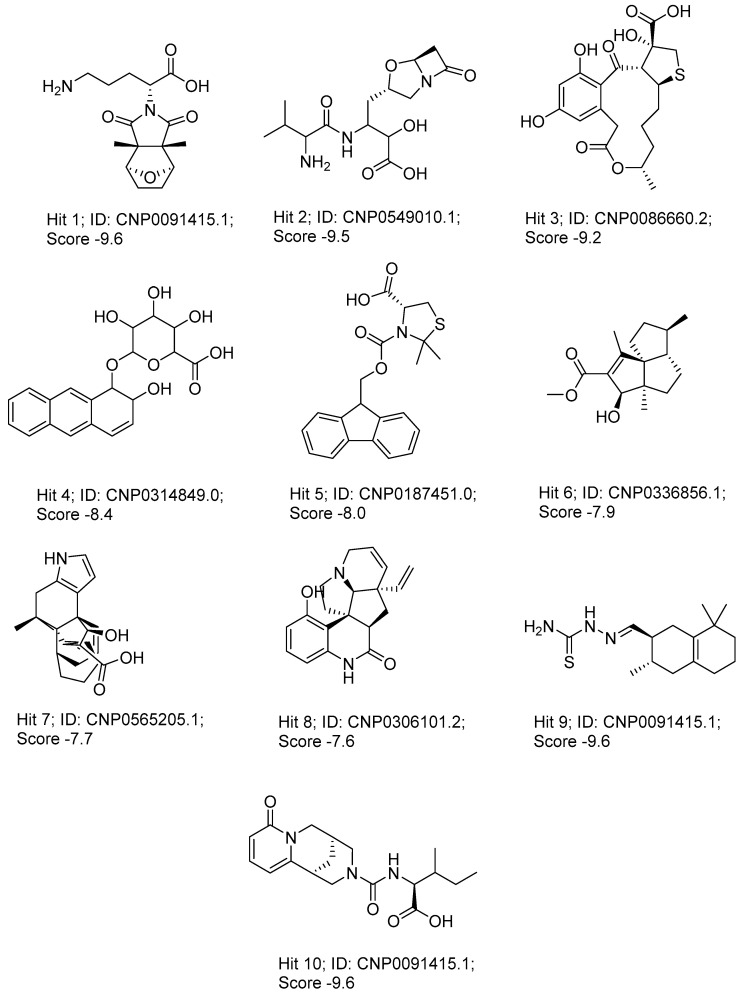

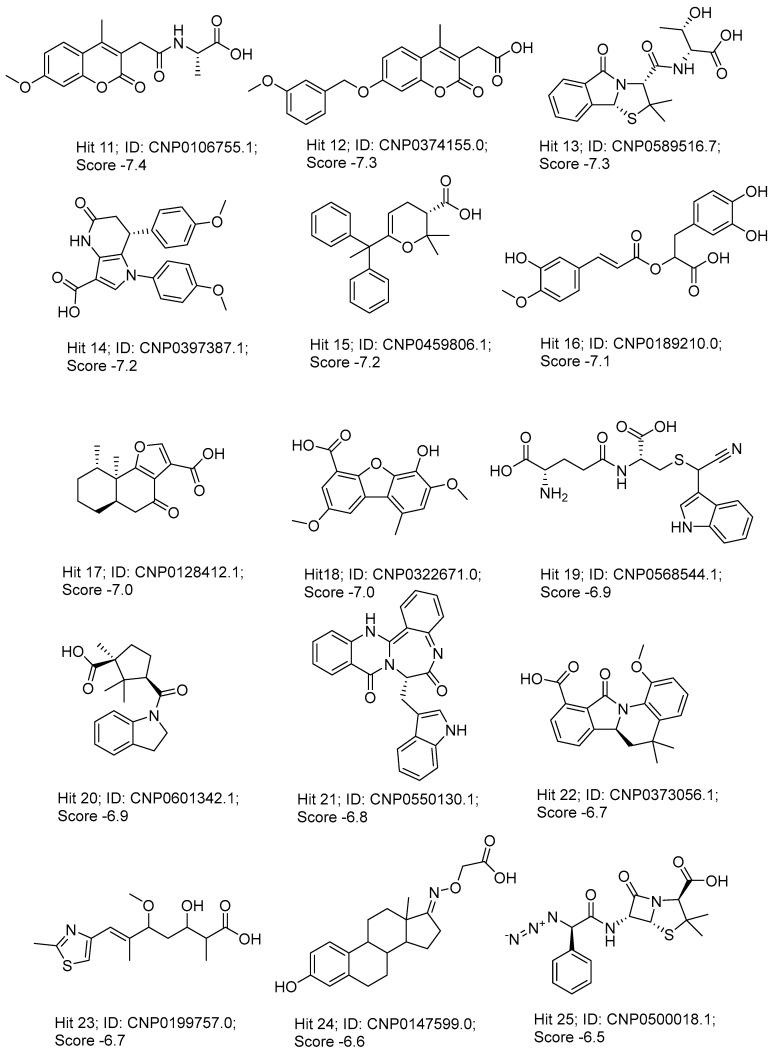

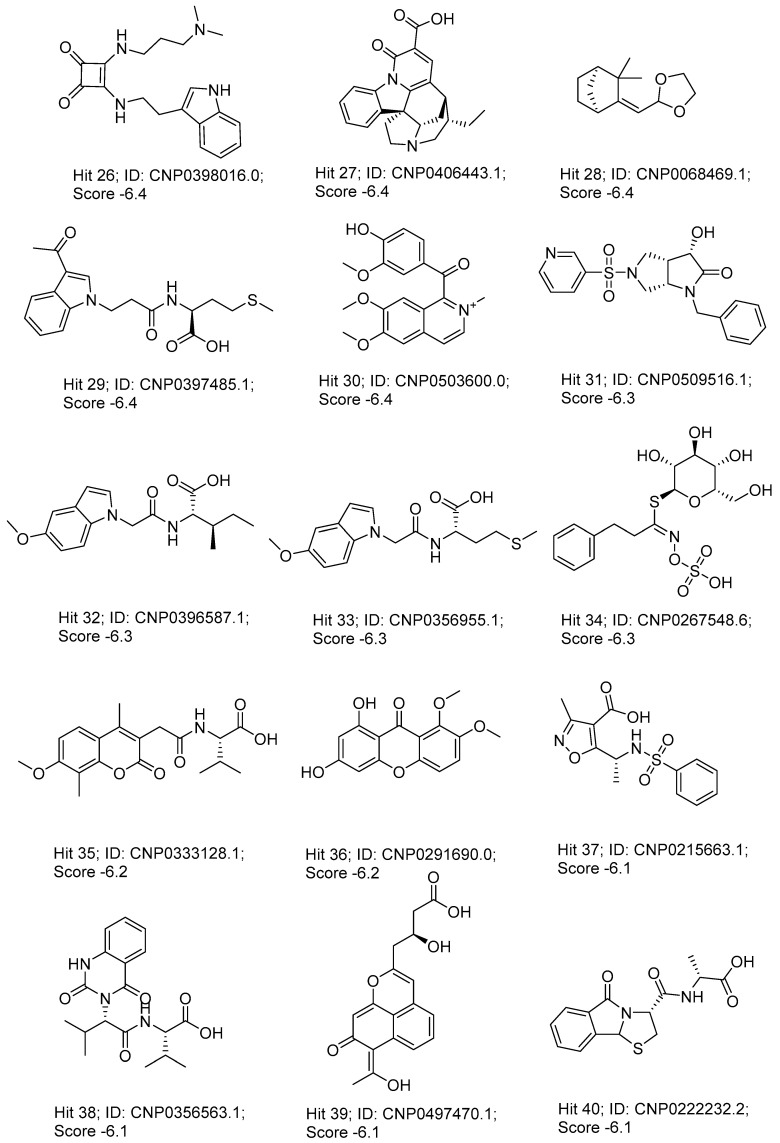

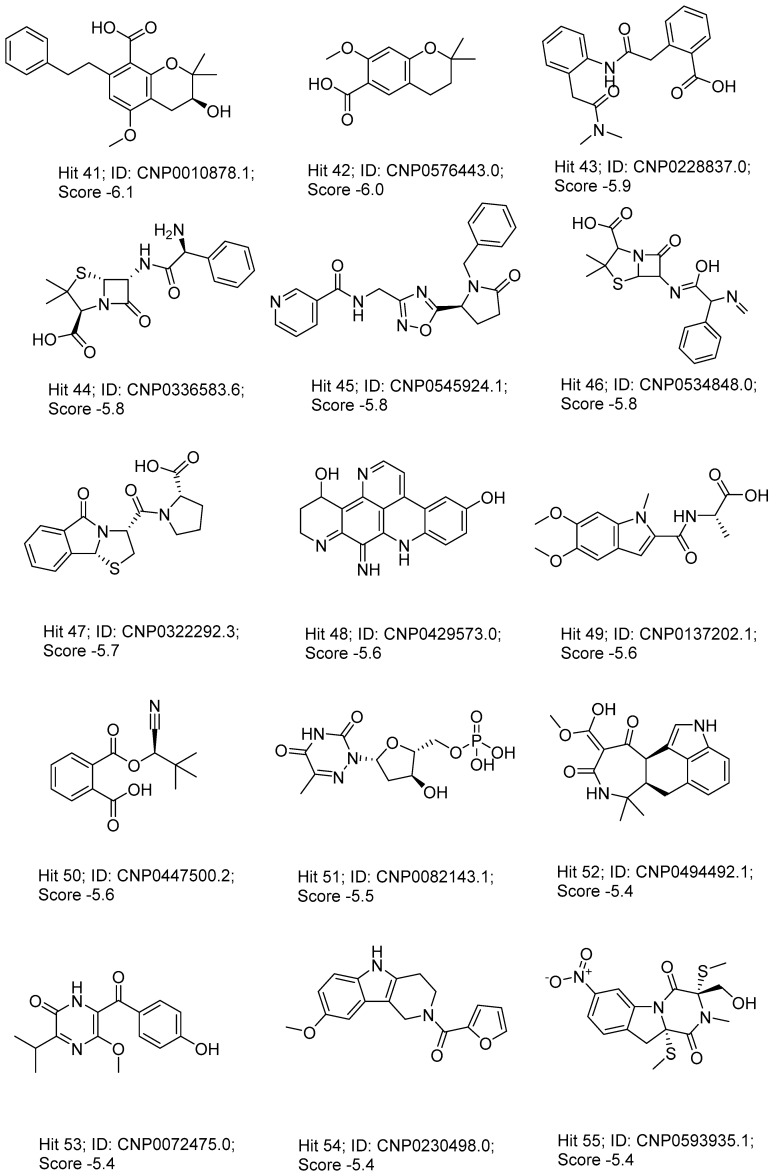

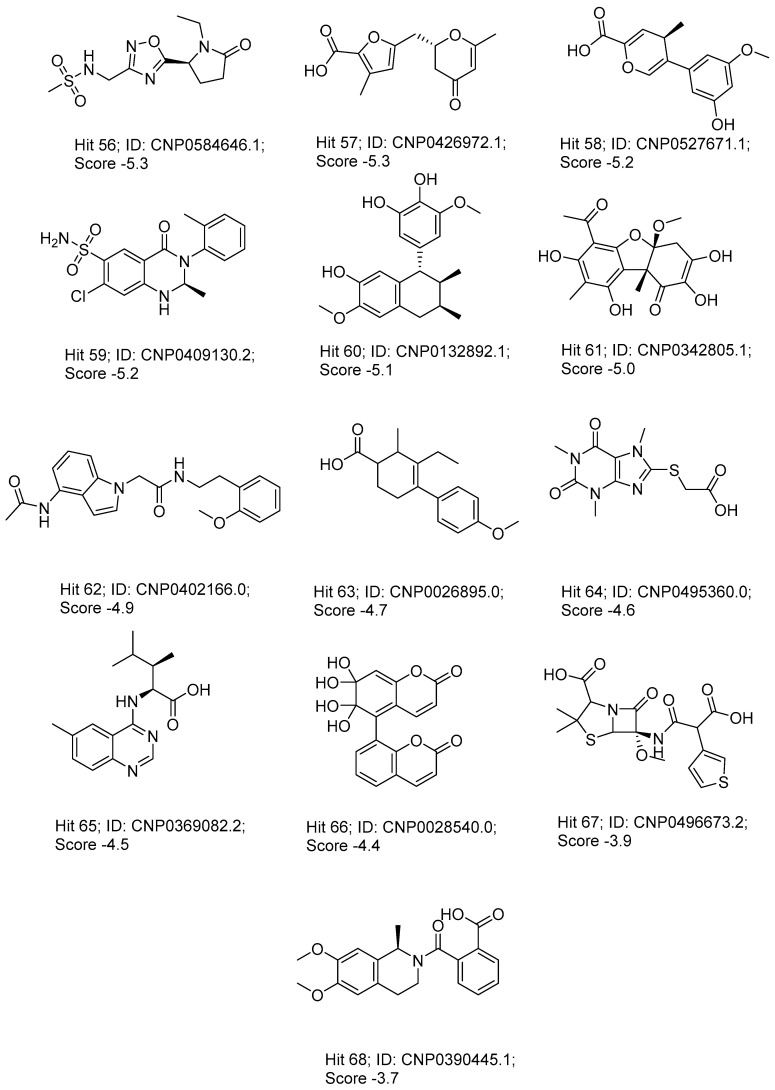
Chemical structures of identified novel scaffolds for GLP-1 positive allosteric modulation with their hit no (hits are arranged according to their XP/Docking score), Coconut ID, and XP/Docking score.

Novel scaffolds for potential GLP-1 positive allosteric modulation were identified from the final XP hits as well as from the original SP and XP docking shortlists after docked poses filtration and XP redocking and MM-GBSA calculations (Figure 12). Those final hits were structurally analyzed to identify common structural scaffolds. These scaffolds would serve as lead compounds to design and develop new GLP-1 allosteric modulators. The list encompassed a variety of compounds from different natural products classes including β-lactams, alkaloids, steroids, sesquiterpenoids, macrolides, and polyketides that possess diverse chemical scaffolds. Peptidomimetic scaffolds were the most frequent scaffolds among the identified hits (36 compounds out of 68 compounds). In these compounds, the peptide moiety was attached to various chemical entities including indole derivatives (hits; **1**, **13**, **14**, **19**, **20**, **22**, **27**, **29**, **32**, **33**, **40**, **47**, **49**, **52**, **54**, **55**, **62**), coumarin derivatives (hits; **11**, **12**, **35**, **39**, **42**), azines and benzazines derivatives (hits; **8**, **10**, **14**, **38**, **53**, **59**, **65**, **68**), and azoles derivatives (hits; **23**, **45**, **56**). The peptide moiety was also found to be incorporated in a cyclic structure including hits; **14**, **21**, **22**, **27**, **52**, **53**, **55**, **59** as well as a chain structure including hits; **1**, **2**, **5**, **10**, **11**, **12**, **13**, **19**, **20**, **25**, **29**, **32**, **33**, **35**, **37**, **38**, **40**, **43**, **44**, **45**, **46**, **47**, **49**, **62**, **64**, **65**, **67**, **68**. β-lactams which could also be considered as cyclic peptidomimetic structure were among the most frequently identified hits (hits; **2**, **25**, **44**, **46**, **67**). A significant number of coumarin derivatives were also identified including hits; **36**, **39**, **41**, **42**, **66**. Sulfonamide derivatives were represented by five hits including hits; **31**, **34**, **37**, **56**, **59**. In conclusion, the structure analysis of the identified hits revealed common structural scaffolds including peptidomimetics, β-lactams, coumarins and sulfonamides, among which peptidomimetics possessed the most significant frequency especially in indole and coumarin cores.

### 3.6. ADMET and Drug-Likeness Profiling

Some interesting features were shown for the top ten hits from the ADMET predicted calculations that influence pharmacokinetic and pharmacodynamic properties (Table 4). Water solubility was highly variable among the hits, with the lowest for hit compound **9** at −4.41 log mol/L, which may indicate potential formulation challenges. Intestinal absorption is notably higher for compounds **5** to **9**, exceeding 90%, which suggests that these molecules have a favorable bioavailability profile. On the other hand, poor absorption was observed with compounds **2** and **4**, probably due to their low solubility and permeability. In the Caco-2 permeability test, compound **9** was identified as the most permeable, with a log Papp of 1.376, further supporting its potential for effective gastrointestinal absorption.

Compounds **8** and **9** may cross the blood–brain barrier considering log BB > 0 given the data for BBB permeability distribution, thus standing out as candidates for CNS-targeting therapies. However, the value for CNS permeability logs PS for all compounds remained low, reflecting restricted access to the tissues of the central nervous system. compound **5** showed the most favorable CNS permeability characteristics, recorded at −2.132 log PS. These results emphasize the importance of refining the distribution parameters during the lead optimization phase, depending on the target of the intended therapy.

Metabolic interactions with the cytochrome P450 enzymes are variable amongst the compounds. Notably, compound **8** was a substrate of CYP2D6 and CYP3A4, possibly suggesting potential for metabolic interactions. The remaining compounds interacted considerably less with the enzymes, and in most instances, one would not predict clinically significant drug–drug interactions. Compound **9** also inhibited CYP2C19 and may affect its metabolism; in vivo investigation is required.

The excretion analysis, judged from total clearance parameters, identified six exhibiting a high clearance value of 1.101 log mL/min/kg; this outcome indicates a possibly short half-life. However, compound **9** revealed negative clearance values, suggesting a possible accumulation and a long systemic exposure. Renal excretion studies revealed that compounds **6** and **8** are substrates for renal OCT2, indicating that active transport plays a primary role in their elimination processes.

Toxicity analyses revealed that all the hit compounds expressed no significant toxicity since none were AMES toxic nor inhibited the hERG channels. However, compounds **1**, **2**, **3**, **5**, and **10** were hepatotoxic, which should be well considered in further stages of development. The maximum tolerated dose was predicted; the highest tolerance was for compound **2**, calculated as 1.50 log mg/kg/day, while the lowest was for 8.

The selected molecules in this study were diverse in their physicochemical, drug-likeness profiles, and medicinal chemistry attributes (Table 5). The MW of the compounds ranged between 264.36 and 410.44 g/mol, hence within the acceptable threshold for small molecules that would enhance permeability and good bioavailability. Lipophilicity was represented by log P values ranging from −1.40 to 3.37. This indicated an appropriate range of hydrophilic–lipophilic properties, supporting the solubility and membrane permeability of most compounds. The number of hydrogen bond acceptors and donors corresponded to Lipinski’s Rule of Five, showing that the structures would be in compliance with the criteria for drug-likeness. At the same time, rotatable bonds and molar refractivity values support these molecules for conformational flexibility and molecular interaction.

From a drug-likeness perspective, all the compounds passed Lipinski’s rule without violation and would thus be orally bioavailable. Veber and Egan showed some alerts due to high topological polar surface area (TPSA), above the threshold in some cases. This minor deviation does not disturb the overall good bioavailability score in the dataset, as the majority of the molecules showed bioavailability scores above 0.55. Although the Ghose and Muegge filters also occasionally violated other parameters, such as XLOGP3 and weight, these were molecule-specific and hence too weak to overpower their overall potential to be good candidates.

Medicinal chemistry analysis showed that none of the compounds had any PAINS alerts; hence, none of these compounds was likely to show false-positive activity upon high-throughput screening. Brenk alerts were seen for some of the molecules; these had structural features like phthalimide which may need further optimization. It was noticed that more than half of these molecules congregate under lead-likeness criteria, while a few exceptions took values above the threshold in rotatable bonds and/or molecular weight. Synthetic accessibility scores spanned a medium range from 3.92 to 5.58, reflecting the fact that it would not result in considerable problems.

## 4. Conclusions

We performed a virtual screening of 695,133 natural products for GLP-1 positive allosteric modulation. The initial database was filtered to ensure oral bioavailability and drug-likeness as well as shape similarity to the crystal ligand. The final database was then screened against the GLP-1 allosteric binding site using three different docking protocols including HTVS, SP, and XP. The results identified the 10 best hits from the initial round, and an additional 58 hits from a second XP round after filtering the conformational poses of the initial XP and SP shortlists based on identified experimental binding criteria. The MM-GBSA were calculated for the 68 hits and demonstrated favorable binding interactions as evident from the exclusively negative binding energies. Molecular dynamics further substantiated the favorable binding profile of the selected hits. The results also highlighted the importance of other residues in the active site that might be crucial for the allosteric binding including Ser 206, Glu 138, and Asp 198. The results identified several hits with previously reported antidiabetic and anti-obesity effects including Sesquiterpenoids, Coumarins, and Phenylpropanoids, NP classes. We were also able to shortlist some suitable compounds for repurposing including Valclavam, Cyclothiocurvularin B, Azidocillin, Ampicillin, Metampicillin, and Timocillin.

The current study also suggests, after thorough structural analysis, that peptidomimetics are preferential scaffolds for GLP-1 positive allosteric modulation. The most common structural features included di- or tri-peptide structure within indole or coumarin cores. These findings, with the proper design of the peptide portion and the proper selection of the non-peptide moiety, may represent attractive lead compounds for preclinical and clinical development.

## Figures and Tables

**Table 1:** Structure, NP class, Coconut ID, XP score, and MM-GBSA of the top 10 hits.

Hit No. *	Structure	NP Class	Coconut Id	XP Score	MM-BBSA DG Bind **
1	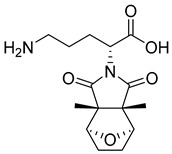	Alkaloid	CNP0091415.1	−9.628	−40.12
2	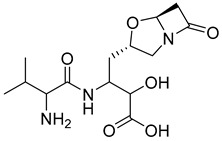	β-lactams	CNP0549010.1	−9.525	−38.18
3	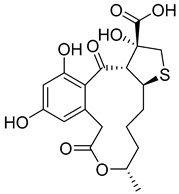	Macrolides	CNP0086660.2	−9.194	−39.22
4	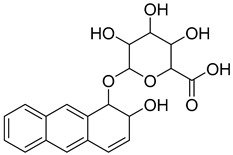	Polyketides	CNP0314849.0	−8.384	−40.01
5	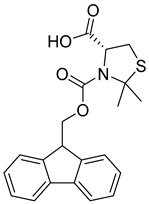	NA	CNP0039190.2	−8.23	−48.15
6	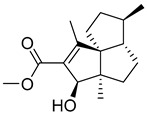	Sesquiterpenoids	CNP0261672.2	−8.121	−46.68
7	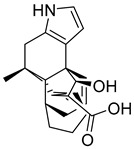	Steroids	CNP0336856.1	−7.958	−32.37
8	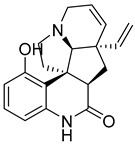	Alkaloids	CNP0380974.1	−7.921	−50.75
9	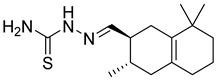	Sesquiterpenoids	CNP0565205.1	−7.698	−46.76
10	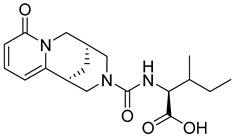	Alkaloids	CNP0306101.2	−7.57	−46.42

* Hits are arranged according to their XP score. ** Calculated in kJ/mol.

**Table 2:** Binding residues and binding interactions of top 10 hits with GLP-1 allosteric site.

Hit No. *	Key Binding Residues and Binding Interactions with GLP-1 Receptor **			Key Binding Residues and Binding Interactions with GLP-1 Peptide **			Additional Binding Residues and Binding Interactions with GLP-1 Receptor **		
	Leu 142	Tyr 145	Lys 202	Phe 12	Val 16	Leu 20	Ser 206	Glu 138	Asp 198
1	V	V	S	V	V	V	H	S	V
2	V	V	S	V	V	V	H	S	V
3	V	H	H	V	V	V	V	V	H
4	V	H	S	V	V	V	V	V	H
5	V	V	S	V	V	V	H	V	V
6	V	V	V	V	V	V	V	V	H
7	V	V	S	V	V	V	V	V	V
8	V	C	V	V	V	V	V	-	H
9	V	V	V	V	V	V	V	H	V
10	V	H	S	V	V	V	V	V	V

* Hits are arranged according to their XP score. ** Calculated in kJ/mol.

**Table 3:** Key binding residues and binding interactions of initial XP and SP hits with GLP-1 allosteric site.

Hit No. *	Coconut ID	Key Binding Residues and Binding Interactions with GLP-1 Receptor **			Key Binding Residues and Binding Interactions with GLP-1 Peptide **			Binding Calculations	
		Leu 142	Tyr 145	Lys 202	Phe 12	Val 16	Leu 20	XP Score	MM-GBSA DG Bind ***
**11**	CNP0106755.1	V	H	H	V	V	V	−7.361	−37.91
**12**	CNP0374155.0	V	P	S	V	V	V	−7.314	−42.18
**13**	CNP0589516.7	V	H	H	V	V	V	−7.285	−39.36
**14**	CNP0397387.1	V	V	H	V	V	V	−7.224	−52.77
**15**	CNP0459806.1	V	V	H	V	V	V	−7.177	−40.16
**16**	CNP0189210.0	V	H	H, S	V	V	V	−7.128	−33.88
**17**	CNP0128412.1	V	V	H, S	V	V	V	−7.01	−36.1
**18**	CNP0322671.0	V	P	H, S	V	V	V	−6.974	−39.96
**19**	CNP0568544.1	V	H	H	V	V	V	−6.928	−30.82
**20**	CNP0601342.1	V	V	H, S	V	V	V	−6.896	−36.72
**21**	CNP0550130.1	V	P	H	V	V	V	−6.745	−50
**22**	CNP0373056.1	V	P	H	V	V	V	−6.673	−38.72
**23**	CNP0199757.0	V	V	H, S	V	V	V	−6.661	−43.63
**24**	CNP0147599.0	V	P	H, S	V	V	V	−6.582	−38.88
**25**	CNP0500018.1	V	H	H, S	V	V	V	−6.548	−37.05
**26**	CNP0398016.0	V	P	V	V	V	V	−6.444	−40.23
**27**	CNP0406443.1	V	C	S	V	V	V	−6.417	−35.03
**28**	CNP0479169.0	V	C	H, S	V	V	V	−6.387	−48.67
**29**	CNP0397485.1	V	V	H	V	V	V	−6.383	−28.54
**30**	CNP0503600.0	V	C	S	V	V	V	−6.353	−46.59
**31**	CNP0509516.1	V	P	H	V	V	V	−6.294	−43.08
**32**	CNP0396587.1	V	V	S	V	V	V	−6.29	−33.45
**33**	CNP0356955.1	V	V	H, S	V	V	V	−6.288	−30.24
**34**	CNP0267548.6	V	P	H, S	V	V	V	−6.279	−42.9
**35**	CNP0333128.1	V	H	H	V	V	V	−6.21	−40.1
**36**	CNP0291690.0	V	V	V	V	V	V	−6.2	−35.25
**37**	CNP0215663.1	V	V	S	V	V	V	−6.111	−30.42
**38**	CNP0356563.1	V	P	S	V	V	V	−6.09	−43.02
**39**	CNP0497470.1	V	V	H, S	V	V	V	−6.09	−33.34
**40**	CNP0222232.2	V	V	S	V	V	V	−6.075	−37.67
**41**	CNP0010878.1	V	P	S	V	V	V	−6.069	−44.52
**42**	CNP0576443.0	V	P	S	V	V	V	−5.952	−29.69
**43**	CNP0228837.0	V	V	S	V	V	V	−5.911	−34.97
**44**	CNP0336583.6	V	H	S	V	V	V	−5.839	−31.27
**45**	CNP0545924.1	V	V	C	V	V	V	−5.815	−38.99
**46**	CNP0534848.0	V	P	H, S	V	V	V	−5.804	−45.32
**47**	CNP0322292.3	V	V	H, S	V	V	V	−5.742	−44.64
**48**	CNP0429573.0	V	V	S	V	V	V	−5.644	−13.02
**49**	CNP0137202.1	V	V	H, S	V	V	V	−5.617	−26.19
**50**	CNP0447500.2	V	V	S	V	V	V	−5.548	−30.9
**51**	CNP0082143.1	V	V	S	V	V	V	−5.521	−23.5
**52**	CNP0494492.1	V	V	H	V	V	V	−5.438	−34.27
**53**	CNP0072475.0	V	H	H, S	V	V	V	−5.437	−24.42
**54**	CNP0230498.0	V	V	C	V	V	V	−5.423	−31.45
**55**	CNP0593935.1	V	V	S	V	V	V	−5.401	−41.93
**56**	CNP0584646.1	V	V	V	V	V	V	−5.306	−26.65
**57**	CNP0426972.1	V	V	S	V	V	V	−5.271	−28.24
**58**	CNP0527671.1	V	P	S	V	V	V	−5.231	−29.94
**59**	CNP0409130.2	V	H	H	V	V	V	−5.205	−47.38
**60**	CNP0132892.1	V	V	V	V	V	V	−5.107	−35.67
**61**	CNP0342805.1	V	V	S	V	V	V	−5.047	−21.43
**62**	CNP0402166.0	V	P	H, C	V	V	V	−4.929	−45.77
**63**	CNP0026895.0	V	H	S	V	V	V	−4.734	−33.7
**64**	CNP0495360.0	V	P	S	V	V	V	−4.643	−26.87
**65**	CNP0369082.2	V	V	H	V	V	V	−4.49	−30.31
**66**	CNP0028540.0	V	V	V	V	V	V	−4.4	−27.38
**67**	CNP0496673.2	V	P	S	V	V	V	−3.917	−25.68
**68**	CNP0390445.1	V	P	H, S	V	V	V	−3.685	−33.48

* Hits are arranged according to their XP score. ** H: Hydrogen bond, S: Salt bridge, P: π-π stacking, C: π-Cation, V: van der Waals. *** Calculated in kJ/mol.

**Table 4:** ADMET profiling of the best 10 hits.

ADMET Parameters	1	2	3	4	5	6	7	8	9	10
Absorption										
Water solubility (log mol/L)	−1.88	−2.24	−3.1	−2.01	−4.12	−3.561	−2.929	−3.808	−4.441	−2.97
Caco2 permeability (log Papp in 10^−6^ cm/s)	0.14	−0.31	−0.17	−0.49	0.67	1.337	1.027	1.21	1.376	0.108
Intestinal absorption (human) (% Absorbed)	42.81	32.3	45	25.50	93.15	95.325	96.606	96.34	91.364	61.309
P-glycoprotein substrate (Yes/No)	NO	Yes	Yes	Yes	Yes	No	No	Yes	No	No
Distribution										
BBB permeability (log BB)	−0.71	−1.007	−1.38	−1.09	−0.04	−0.007	−0.313	0.142	0.098	−0.953
CNS permeability (log PS)	−3.18	−4.15	−3.80	−3.92	−2.132	−2.176	−2.333	−2.135	−3.22	−3.414
Metabolism										
CYP2D6 substrate (Yes/No)	No	No	No	No	No	No	No	Yes	No	No
CYP3A4 substrate (Yes/No)	No	No	No	No	Yes	Yes	Yes	Yes	No	No
CYP1A2 inhibitor (Yes/No)	No	No	No	No	Yes	No	No	No	No	No
CYP2C19 inhibitor (Yes/No)	No	No	No	No	No	No	No	No	Yes	No
CYP2C9 inhibitor (Yes/No)	No	No	No	No	No	No	No	No	No	No
CYP2D6 inhibitor (Yes/No)	No	No	No	No	No	No	No	No	No	No
CYP3A4 inhibitor (Yes/No)	No	No	No	No	No	No	No	No	No	No
Excretion										
Total Clearance (log mL/min/kg)	0.93	1.04	0.225	0.524	0.196	1.101	0.506	0.762	−0.374	1.05
Renal OCT2 substrate (Yes/No)	No	No	No	No	No	Yes	No	Yes	No	No
Toxicity										
AMES toxicity (Yes/No)	No	No	No	No	No	No	No	No	No	No
Max. tolerated dose (human) (log mg/kg/day)	0.89	1.50	0.847	−0.224	0.039	−0.465	0.2	−0.741	0.171	0.906
hERG I inhibitor (Yes/No)	No	No	No	No	No	No	No	No	No	No
Hepatotoxicity (Yes/No)	Yes	Yes	Yes	No	Yes	No	No	No	No	Yes

**Table 5:** Physicochemical properties, drug-likeness, and medicinal chemistry prediction of the best 10 hits.

Property	1	2	3	4	5	6	7	8	9	10
Physicochemical properties										
Molecular Weight (g/mol)	310.35	329.353	410.44	388.37	383.46	264.36	337.41	308.37	279.44	347.41
LogP	1.76	−1.40	1.25	0.16	3.37	2.94	2.78	2.18	3.15	1.38
#Acceptors	6	6	8	8	4	3	3	3	1	4
#Donors	2	4	4	5	1	1	3	2	2	2
#Heavy atoms	22	23	28	28	27	19	25	23	19	25
#Arom. heavy atoms	0	0	6	10	12	0	5	6	0	6
Fraction Csp3	0.80	0.79	0.53	0.35	0.33	0.81	0.57	0.42	0.73	0.61
#Rotatable bonds	5	7	1	3	5	2	1	1	3	6
Molar refractivity	80.13	81.92	101.34	97.02	109.12	74.14	93.61	95.75	86.24	97.55
TPSA (Å^2^)	109.93	142.19	166.66	136.68	92.14	46.53	73.32	52.57	82.50	91.64
Drug-likeness										
Lipinski alert	Yes; 0 violation	Yes; 0 violation	Yes; 0 violation	Yes; 0 violation	Yes; 0 violation	Yes	Yes	Yes	Yes	Yes
Ghose	Yes	No; 1 violation: WLOGP < −0.4	Yes	Yes	Yes	Yes	Yes	Yes	Yes	Yes
Veber	Yes	No; 1 violation: TPSA > 140	No; 1 violation: TPSA > 140	Yes	Yes	Yes	Yes	Yes	Yes	Yes
Egan	Yes	No; 1 violation: TPSA > 131.6	No; 1 violation: TPSA > 131.6	No; 1 violation: TPSA > 131.6	Yes	Yes	Yes	Yes	Yes	Yes
Muegge	No; 1 violation: XLOGP3 < −2	No; 1 violation: XLOGP3 < −2	No; 1 violation: TPSA > 150	Yes	Yes	Yes	Yes	Yes	Yes	Yes
Bioavailability Score	0.55	0.55	0.11	0.56	0.56	0.55	0.85	0.55	0.55	0.56
Medicinal chemistry										
PAINS	0	0	0	0	0	0	0	0	0	0
Brenk	1 alert: phthali-mide	0	0	0	0	0	1	1	3	0
Leadlikeness	Yes	No; 1 violation: Rotors > 7	No; 1 violation: MW > 350	No; 1 violation: MW > 350	No; 2 violations: MW > 350, XLOGP3 > 3.5	Yes	Yes	Yes	Yes	Yes
Synthetic accessibility	3.92	4.48	5.15	5.09	4.23		5.00	5.58	4.74	4.74

## Data Availability

In silico drug experiments using molecular docking to target GLP-1 receptor (PDB ID: 6VCB) were obtained from the Research Collaboratory for Structural Bioinformatics (RCSB) Protein Data Bank (PDB). PDB DOI: “https://doi.org/10.2210/pdb6VCB/pdb (accessed on 20 June 2024)”. Natural products database was downloaded from the Coconut website: “https://coconut.naturalproducts.net/download (accessed on 20 June 2024)”. All data generated or analyzed during this study are included in this published article.

## Supplementary Materials

The following supporting information can be downloaded at: https://www.mdpi.com/article/10.3390/pharmaceutics16121607/s1, MM-GBSA Data for the hit compounds.
